# Smart Injectable Self-Setting Monetite Based Bioceramics for Orthopedic Applications

**DOI:** 10.3390/ma11071258

**Published:** 2018-07-22

**Authors:** Naresh Koju, Prabaha Sikder, Bipin Gaihre, Sarit B. Bhaduri

**Affiliations:** 1 Department of Mechanical Industrial and Manufacturing Engineering, The University of Toledo, Toledo, OH 43606, USA; naresh.koju@gmail.com (N.K.); sarit.bhaduri@gmail.com (S.B.B.); 2 Department of Bioengineering, the University of Toledo, Toledo, OH 43606, USA; bipin.gaihre@rockets.utoledo.edu

**Keywords:** smart, calcium phosphate, monetite, barium titanate, bioceramics, orthopedics

## Abstract

The present study is the first of its kind dealing with the development of a specific bioceramic which qualifies as a potential material in hard-tissue replacements. Specifically, we report the synthesis and evaluation of smart injectable calcium phosphate bone cement (CPC) which we believe will be suitable for various kinds of orthopedic and spinal-fusion applications. The smart nature of this next generation orthopedic implant is attained by incorporating piezoelectric barium titanate (BT) particles into monetite-based (dicalcium phosphate anhydrous, DCPA) CPC composition. The main goal is to take advantage of the piezoelectric properties of BT, as electromechanical effect plays a vital role in fracture healing at the defect site and bone integration with the implant. Furthermore, radiopacity of BT would help in easy detection of the CPC presence at the fracture site during surgery. Results reveal that BT addition favors important properties of bone cement such as good compressive strength, injectability, bioactivity, biocompatibility, and even washout resistance. Most importantly, the self-setting nature of the bone cements are not compromised with BT incorporation. The in vitro results confirm that the developed bone-cement abides by the standard orthopedic requirements making it apt for real-time prosthetic materials.

## 1. Introduction

This paper reports the development of a new generation, smart, injectable calcium phosphate cement (CPC) for orthopedic applications. The “smart” aspect of the compositions is achieved by incorporating piezoelectric materials into the cement. Piezoelectric materials develop surface charges when compressive stresses are applied to them. Research over the last five decades has unequivocally shown that the important bone constituents such as apatite and collagen, both show piezoelectric behavior [1,2,3,4,5,6,7,8]. This coupled electromechanical phenomenon has an important effect on healing of an orthopedic defect as well as integration of bone with an implant. Park et al. conducted in vivo experiments trying to understand the effect of applied electric field in osseointegration of implants [9,10,11,12]. The experiments clearly showed the beneficial effects of an applied electric field. Subsequently, they hypothesized that a piezoelectric implant can deliver the same beneficial effect by generating an electric field in situ without the application of an external field. Barium titanate (BaTiO_3_, BT), one of the most extensively studied piezoelectric material was the obvious candidate of choice for implantation. In the early eighties, Park et al. reported that dense sintered BT implants were able to stimulate bone growth in dog femurs [13,14]. The implants were stable in the dog for several months and did not cause cytotoxicity. These initial experiments showed the effective applications of piezoelectric BT in orthopedics.

However, since those early reports of BT as an implant material, the field did not progress further in terms of materials development. The next phase of development took place after almost two decades with the development of hydroxyapatite–barium titanate (HA–BT) composites. Jianqing et al. developed composites by using conventional sintering and polarized them before implanting into dog jawbones [15]. A clear rationale was not provided as to why there was the need to develop composites, in view of the fact that both HA and BT are piezoelectric, biocompatible but not bioactive. In other words, the constituents did not possess complementary properties. The results showed accelerated bone formation kinetics around HA–BT implants as compared to pure HA [15]. Progressing along the same strategical avenue, Dubey et al. and Prakasam et al. fabricated BT infused HA composites via multi and single-stage spark plasma sintering (SPS) respectively and the results yielded good biomechanical and electrical properties [16,17]. Furthermore, when surface charge was generated by electrical poling of such composites, higher cell proliferation was observed on the negatively charged surfaces [18]. In vivo studies revealed no cytotoxic reactions from HA–BT (containing 40 wt % BT) when implanted into the right knee joints of mice [19]. This finding is comparable to that which Park et al. reported earlier. Even porous nanophase HA–BT composites were proven to be potential candidates in the fabrication of piezoelectric orthopedic implants [20]. All of these materials were fabricated via a powder metallurgical process using different types of sintering techniques.

An important question explored in this respect is to evaluate the effect of BT addition into injectable self-setting orthopedic cement compositions. This would eliminate the requirement of the high temperature sintering process and the fabrication of simple shapes, and enable the cement to be injected at the defect site using a minimally invasive procedure. To the best of our knowledge, Carrodeguas et al. reported the preparation of an injectable bone cement consisting of poly (methyl methacrylate) (PMMA) matrix mixed with BT or strontium titanate (SrTiO_3_, ST) [21,22]. The main idea was to enhance the radio-opacity of the cement with the incorporation of either BT or ST. The cement was injectable and self-setting besides being radio-opaque. This property helps in monitoring the cement flow under fluoroscopy to avoid leakage. However, PMMA compositions possess the usual drawbacks of setting with high exothermicity besides being inert. Furthermore, no attention was paid to the beneficial effects of piezoelectric properties of BT. Summarizing, this literature search shows that there has been no report of a BT containing smart self-setting bioactive CPC, which incorporates all the beneficial properties of its constituents.

The present study is one of the first of its kind to focus on incorporating BT into monetite-based (dicalcium phosphate anhydrous, DCPA) CPC composition. There are two reasons for choosing the DCPA-based CPC composition. First, commercial CPCs can be mainly classified as apatite and brushite (DCPD) cement [23,24]. DCPD resorbs quickly and transforms into a stable apatite phase after in vivo implantation [25]. Furthermore, the release of orthophosphoric acid during the degradation of DCPD to apatite might cause a certain degree of tissue inflammation [24]. On the other hand, DCPA exhibits a much more stable degradation rate and does not transform into hydroxyapatite (HA), thus increasing the chances of higher bone volume formation [26,27,28,29]. This makes DCPA a perfect CPC candidate. Second, our group has a sustained research thrust in developing CPC compositions, especially DCPA. The present work is the next phase of our CPC research, reporting the development of a novel non-exothermic injectable CPC composition incorporating BT and investigations on its physical, mechanical, electrical properties and their cytocompatibility in vitro. 

## 2. Materials and Methods 

### 2.1. Materials

Barium titanate (IV) (BaTiO_3_, 99.5%, particle size ≤2 µm) and colloidal silica were procured from Sigma–Aldrich (Saint Louis, MO, USA) and used without further modification. The detailed information of colloidal silica is presented in Table 1. Calcium hydroxide (Ca(OH)_2_, >95%), magnesium hydroxide (Mg(OH)_2_, 95%), sodium bicarbonate (NaHCO_3_, >99.7%) and magnesium oxide (MgO, 98%) from Fischer Scientific (Fair Lawn, NJ, USA) and sodium tetraborate decahydrate as known as borax decahydrate (Na_2_B_4_O_7_·10H_2_O, >99.5%) from Alfa Aesar (Ward Hill, MA, USA) were used for the preparation of the cement samples.

### 2.2. Sample Preparation

#### 2.2.1. Premixed Powder (PMP) Preparation 

Preparation of CaP cement (CPC) samples requires a kind of setting solution and premixed powder (PMP). The setting solution was prepared by diluting 78 mL of o-phosphoric acid (H_3_PO_4_, 85%) with 12 mL of Deionized (DI) water placed in an ultrasonic bath, followed by the addition of 12 g sodium bicarbonate. The preparation of the setting solution is critical in material preparation as it influences the setting time of the fabricated bone cement. For a controlled reaction rate, 3 g of NaHCO_3_ was added over an interval of 30 s. The ultrasonic bath was kept running until the setting solution became clear and then was stored in a tightly capped glass bottle. To prepare PMP, initially, 55.2 g of Ca(OH)_2_ and 13.8 g of Mg(OH)_2_ were mixed homogeneously in a household mixer (KitchenAid Classic 275 W, KitchenAid, MI, USA). After few minutes of mixing, the setting solution was slowly poured into the mixer bowl and allowed to run for another 2 min. The mixer was kept running at the lowest speed for the whole time. At the end of 2 min, then formed chunks were taken out into a glass bowl and placed immediately inside a household microwave (Panasonic 1250 W, Panasonic Appliances, Shanghai, China) and irradiated at 10% power level (Level 1) for 6 min. This microwave cycle was repeated several times until the chunks became rock-hard. Finally, the rock-hard cement precursors were ground into powder using a mortar and pestle and PMP fine powder (≥250 µm) was obtained by sieving it through USA Test Sieve (ASTM E11 specification [30]). 

#### 2.2.2. BT Cement Samples Preparation 

Borax decahydrate (4 wt % of PMP), MgO (5 wt % of PMP) and varying amounts of BT (0, 10, 20, 30, 40 wt % of total powders) were added to 5 g PMP and mixed homogeneously. Colloidal silica (liquid) was added to the powder mixture phase using a pipette such that the liquid to powder (L/P) ratio was maintained constant at 0.35 mL/g. The specimen name along with its compositions are presented in Table 2. After addition of colloidal silica, the mixture was vigorously mixed for 60 s using a mortar and pestle. Finally, the formed putty was put into cylindrical molds and prepared for various characterizations.

### 2.3. Setting Time

The initial and final setting time of the smart CPC-x BT (x = wt % of BT) cements were obtained using the Gillmore needle method (ASTM C266-89 [31]). Light and thick needle measures the initial setting time while heavy and thin needle measures the final setting time. The initial and final setting times were recorded when the needle left an indentation depth less than 1 mm on the sample surface. Additionally, respective setting times were measured at an interval of 15 s. 

### 2.4. Mechanical Properties

Smart CPC-x BT samples with dimensions, Ø 12.5 mm and height 7 mm, were prepared for mechanical properties evaluation. Cement samples were allowed to set for 24 h at room temperature. Compression strength testing was carried out on a universal testing machine with the application of 50 kN uniaxial load cell (model 5569, Instron, Norwood, MA, USA). As per the protocol of the American dental association i.e., 0.75 ± 0.25 mm·min^−1^, the crosshead loading rate of 0.5 mm·min^−1^ was set [29].

### 2.5. Physical Characterizations 

#### 2.5.1. X-ray Diffraction Analysis

X-ray diffraction analysis (XRD, Ultima III; Rigaku, The Woodlands, TX, USA) with mono chromated Cu Kα radiation (44 KV, 40 mA) was used to study the phases present in the as-prepared CPC-x BT samples over 2θ range of 10–60°. MD JADE software 2010 (MDI, Livermore, CA, USA) was employed to identify the respective phases.

#### 2.5.2. Fourier Transform Infrared Spectroscopy Analysis

The functional groups present within the samples were identified with Fourier transform infrared spectroscopy (FTIR, UMA-600 Microscope, Varian Excalibur Series, Digilab, Holliston, MA, USA) using an Attenuated total reflection (ATR) diamond crystal. For each sample, 256 scans were performed within the range of 4000 to 700 cm^−1^.

#### 2.5.3. Morphological Observation 

Surface morphology of the samples was examined using scanning electron microscope (SEM, S-4800, Hitachi, Tokyo, Japan). All the samples were mounted on SEM stubs with a copper conducting tape and sputter coated for 90 s before conducting the SEM study. 

### 2.6. Simulated Body Fluid (SBF) Immersion

Smart CPC-x BT samples pellets (Ø 6.4 × 3 mm^3^) were immersed into tightly capped 50 mL autoclaved bottles filled with 30 mL 1.5 × t-SBF. The detailed composition of 1.5 × t-SBF is shown in Table 3 [32]. The bottles containing the SBF immersed samples were placed in a water bath at 37 °C for 7 days. Moreover, the SBF was replenished every other day in order to keep the ionic compositions constant. After the time period, samples were cleaned under flowing distilled water and thoroughly dried in an air-convection furnace at 60 °C for further characterization under SEM.

### 2.7. Injectability

Colloidal silica was added to Na_2_B_2_O_7_·10H_2_O, MgO, and varying amounts of PMP and BT to form putty-like materials which were put into syringes (BD Biosciences, San Jose, CA, USA) with a nozzle diameter of 1.36 mm. The nozzle diameter used in the present work lies between gauge 15 (1.449 mm) and gauge 16 (1.291 mm) and is smaller than the actual needle size used in clinical cement injection [33]. After loading the putty, the plunger of the syringe was propelled manually to examine the injectability of the as-prepared CPC-x BT cement samples. The samples were considered to have certifiable injectability if the cement did not remain inside the syringe during injection. 

### 2.8. Washout Resistance Test

As per the Takagi et al. protocols, different composition of cement pastes were prepared, loaded into syringes and then immediately injected into saliva-like solution (SLS) at 37 °C even before the initial setting time [34]. Immediate injection of cement mimics the real-time orthopedic application and thus is a pragmatic evaluation. SLS was prepared using 1.2 mmol/L calcium chloride (CaCl_2_), 0.72 mmol/L monopotassium phosphate (KH_2_PO_4_), 30 mmol/L potassium chloride (KCl), 50 mmol/L N-2-hydroxyethyl-piperazine-N′-2′-ethane sulfonic acid (HEPES) buffer and finally its pH was adjusted to 7 with 0.1 mol/L NaOH. The sample was considered to pass the washout resistance test if it did not visibly disintegrate in the SLS for 5 min.

### 2.9. Biodegradation

In order to study the in vitro biodegradation rate of smart CPC-x BT samples (Ø 12.5 × 7 mm^3^), 1.5 × t-SBF solution was used as the medium. As per the Wu et al. protocol, cement samples, after they had set for 24 h, were immersed in glass bottles containing 1.5 × t-SBF. The bottles were then placed in a water-bath shaker which maintained 37 °C and 100 rpm. The weight-to-volume ratio of the test specimens were also maintained at 0.2 gm/mL [35,36,37]. For over a time span of 7 days, each and every day the samples were retrieved from the SBF solution, cleaned in deionized water, dried at 60 °C for 2 h, and weighed for any weight loss. The dried coupons were re-immersed into fresh 1.5 × t-SBF solution with repetition of the above-mentioned steps. The biodegradation rate of the coupons at various points of time was calculated using the Equation (1).

(1) $$\mathrm{Weight}\;\mathrm{loss}=\frac{W_{i}-W_{d}}{W_{d}}\times 100\%$$

where, *W_i_* represents initial coupon weight, and *W_d_* represents dried coupon weight after degradation. 

### 2.10. In Vitro Cytocompatibility

#### 2.10.1. WST-1 Assay

OB-6 pre-osteoblast cell line and water-soluble tetrazolium (WST-1) assay were used to study the cytocompatibility of the smart cement samples. This is a colorimetric assay based on the conversion of stable tetrazolium salt into soluble formazan by the cellular mechanism that occurs at the cell surface. Hence, the amount of formazan detected directly relates to the number of metabolically active cells. For this assay, sample pellets (Ø 6.4 × 3 mm^3^) were incubated in complete culture medium for 24 h at 37 °C and 5% CO_2_ in a 24 well plate, following the extraction ratio of 3 cm^2^/mL. In a 96-well plate, 10,000 cells/well were plated and incubated at the same conditions for 24 h. After 24 h, the culture medium on the cell plated 96-well plate was replaced with 100 µL of the extracted or conditioned medium (the medium which contained the specimens for 24 h). The cells were further cultured in the conditioned media for a period of 24 h or 72 h at 37 °C and 5% CO_2_. At specific time points, 10 µL (1:10 ratio) of WST-1 reagent was added to wells containing cells/medium and incubated for 4 h under the same conditions. After 4 h, the formation of water soluble formazan was detected at 450 nm using a SpectraMax 190 microplate reader (Molecular Devices, San Jose, CA, USA). 

#### 2.10.2. Live and Dead Assay

The viability of pre-osteoblasts (OB-6) attached to the samples was imaged using a Cytation 5 cell imaging multi-mode reader (BioTek, Winooski, VT, USA) after staining them with Live/Dead cell viability kit (Thermofisher Scientific, Waltham, MA, USA) at day 5. The samples were first kept under UV light for 15 min followed by incubation with complete cell culture media for 1 h at 37 °C and 5% CO_2_. The OB-6 cells harvested from the cell-culture dishes were then seeded onto the top of the samples at 30,000 cells/sample. In order to allow the proper attachment of the cells to the sample, 200 µL of cell suspension containing 30,000 cells was first added to the samples and incubated at 37 °C and 5% CO_2_ for 3 h. After 3 h, remaining media was added, and the incubation was continued, and the media was changed every third day. On day 5, the cell-sample construct was moved to the new well and washed twice with 1X PBS. The Live/Dead assay solution containing calcein AM and ethidium homodimer (EthD-1) diluted in 1X Dulbecco’s-PBS was added to the samples and incubated for 30 min at 37 °C. The viable cells attached to the samples were indicated by the green fluorescence of calcein which has been enzymatically converted from calcein AM by live cells. Similarly, the dead cells were indicated by the red fluorescence obtained due to the binding of EthD-1 that entered through the ruptured cells to the nucleic acid.

### 2.11. Statistical Analysis

All the experiments were carried out in triplicate for each composition and the results were represented as mean ± SD. Statistical analysis of obtained results was carried out using a one-way analysis of variance (one-way ANOVA). To depict statistically different groups Tukey’s test with *p* < 0.05 was carried out.

## 3. Results

### 3.1. Setting Time

The initial and final setting times for smart CaP cements with different concentrations of BT at a constant L/P ratio of 0.35 mL·g^−1^ are presented in Table 4. No significant variation (*p* > 0.05) were observed except for 10 wt % BT samples. The CPC-10 BT samples displayed significantly (*p* < 0.05) lower final setting time when compared to other formulations.

### 3.2. Mechanical Properties

**Figure 1:**
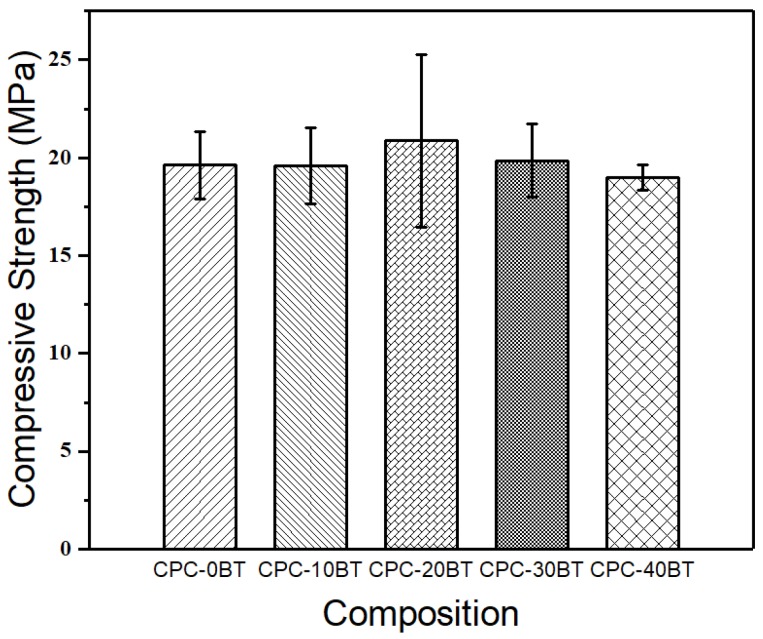
Compressive strengths of different compositions of smart calcium phosphate bone cements (CPCs).

The results of compressive strength of smart CPC samples with varying wt % of BT are displayed in Figure 1. All compositions showed similar compressive strength without any significant differences (*p* > 0.05). This indicates the negligible effect of BT addition on compressive strength of CPC cements up to 40 wt %.

### 3.3. Physical Characterizations

#### 3.3.1. XRD Analysis

**Figure 2:**
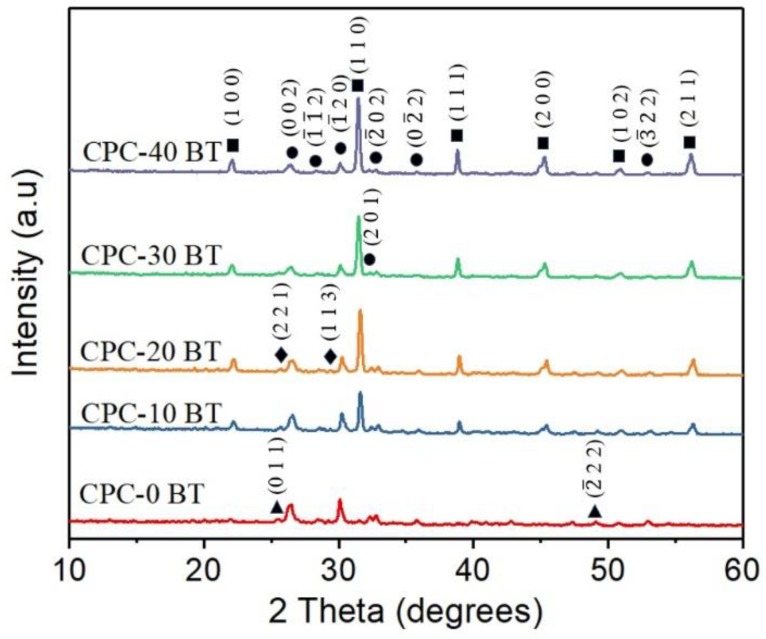
X-ray diffraction (XRD) patterns of CPC with various wt % BaTiO_3_ (‘∎’ represents barium titanate (BT), ‘●’ represents monetite, ‘♦’ represents newberyite and ‘▲’ represents Ca(H_2_PO_4_)_2_).

The self-setting smart CPC-x BT cement samples were crushed into powder used for XRD analysis. The XRD spectra of the respective specimens are shown in Figure 2. All major peaks of CPC-0 BT correspond to monetite (JCPDS PDF# 97-003-8128). With incorporation of BT into PMP, apart from monetite, new peaks corresponding to barium titanate (JCPDS PDF# 97-002-7969) were identified. All the XRD peaks of BT showed much higher crystallinity as compared to monetite. The major peak of BT was present at the 2θ angle of 31.5° along the plane [110], and its intensity intensified with increasing doping percentage. The XRD analysis also picked up small traces of newberyite (MgHPO_4_·3H_2_O) and calcium dihydrogen phosphate (Ca(H_2_PO_4_)_2_). 

#### 3.3.2. FTIR Analysis

**Figure 3:**
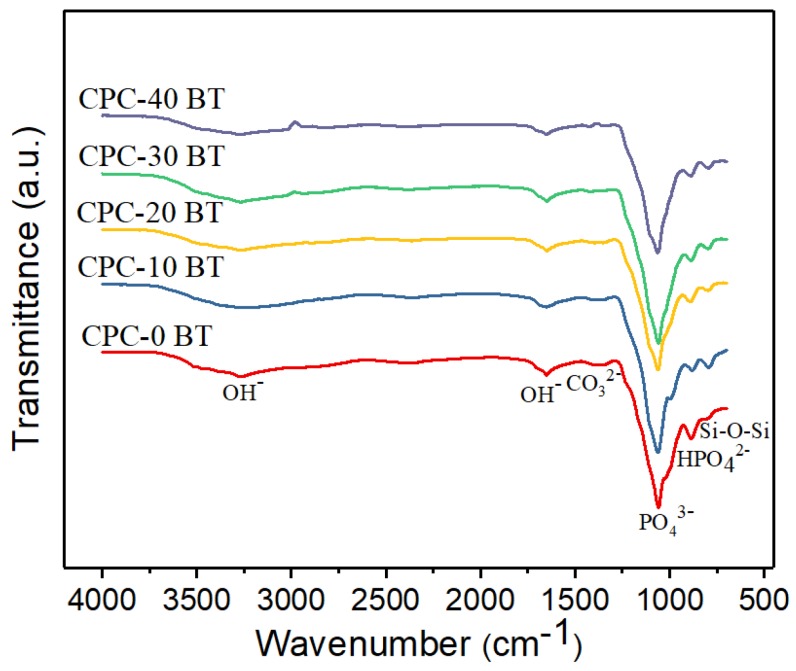
Fourier transform infrared spectroscopy (FTIR) spectra of smart CPC formulation with varying wt % BT.

The FTIR spectra of smart CPCs with different concentration of BT are presented in Figure 3. All samples have similar absorbance spectra with the presence of hydroxyl (OH^−^), phosphate (PO_4_^3−^)/hydrogen phosphate (HPO_4_^2−^), and carbonate (CO_3_^2−^) functional groups. OH^−^ groups are observed in the broad stretching band at 3000–3500 cm^−1^ which corresponds to absorbed water and at 1650 cm^−1^ [37,38]. The CO_3_^2−^ bands are located near 1415 cm^−1^ like all bio­logical apatite [39]. The band at 1020 cm^−1^ accounts for the stretching mode of the P–O bond [40]. The shoulder peaks at 880 and 1060 cm^−1^ can be attributed to the presence of PO_4_^3−^ and/or HPO_4_^2−^. The presence of several visible peaks and shoulder in the region of absorbance spectra of the phosphate region (900–1200 cm^−1^) for all CPC-x BT samples implies the crystallinity of monetite even in the presence of BT [38]. In addition to these functional groups, the stretching band at 790 cm^−1^ corresponding to Si-O-Si (siloxane) group is observed [41].

#### 3.3.3. Morphological Observation of Cements

**Figure 4:**
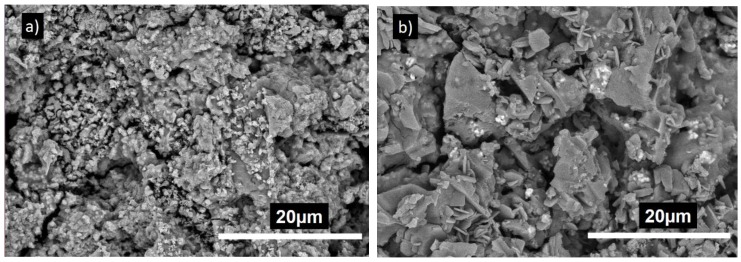

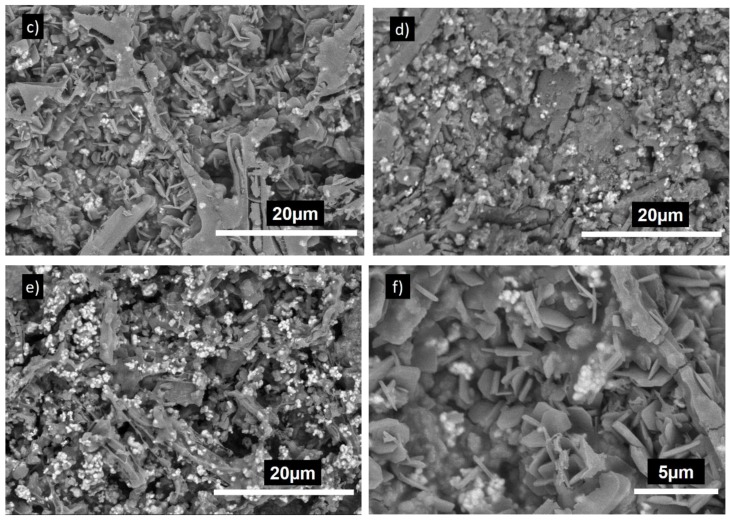
Scanning electron microscopy (SEM) images of (**a**) CPC-0BT, (**b**) CPC-10BT, (**c**,**f**) CPC-20BT, (**d**) CPC-30BT, (**e**) CPC-40BT.

The SEM images of smart CPC-x BT are shown in Figure 4. All the cement specimens were composed of plate-like DCPA crystals [29,40,42]. Back scattered SEM images projected BT particles (Figure 4b–f) as white spherical structures distributed homogeneously all over the cement surface area. The surface morphology revealed highly crystalline DCPA structures in CPC specimen especially with 10 and 20 wt % BT incorporation. Higher magnification image (Figure 4f) shows the morphology of the BT particles assimilated over the CPC matrix. As expected, with increase in BT wt % in the sample composition, the concentration of white agglomerates also increased.

### 3.4. Simulated Body Fluid (SBF) Immersion

**Figure 5:**
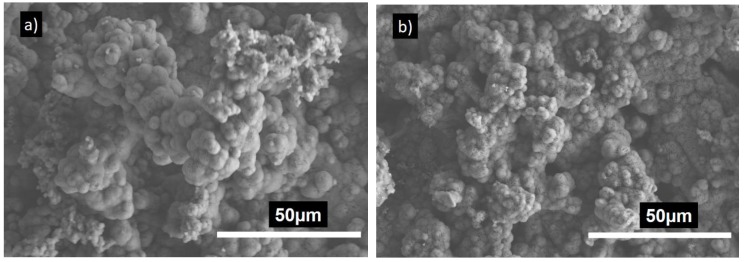

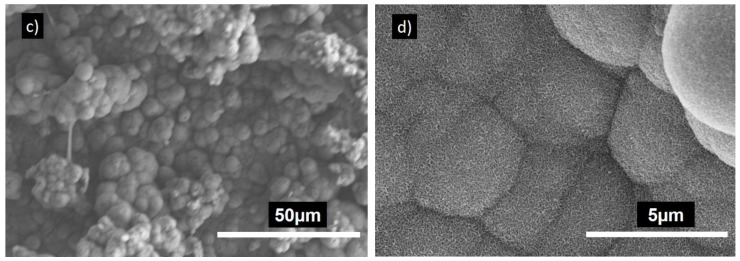
SEM images of (**a**) CPC-0 BT, (**b**,**d**) CPC-20 BT and (**c**) CPC-40 BT after 7 days immersion in 1.5 × simulated body fluid (SBF).

The bioactivity of smart CPC with 0, 20 and 40 wt % BT was examined via an SBF immersion test. After soaking the samples in 1.5 × t-SBF for 7 days, they were characterized by SEM and the results are shown in Figure 5. A uniform dense layer of globular and flower-like structure covered the whole surface of the specimens. With the similarity in morphology they can be identified as apatite. Coatings resulting from SBF immersion usually result in the formation of apatite. Moreover, incorporation of BT did not influence the bioactivity of the samples. 

### 3.5. Injectability

**Figure 6:**
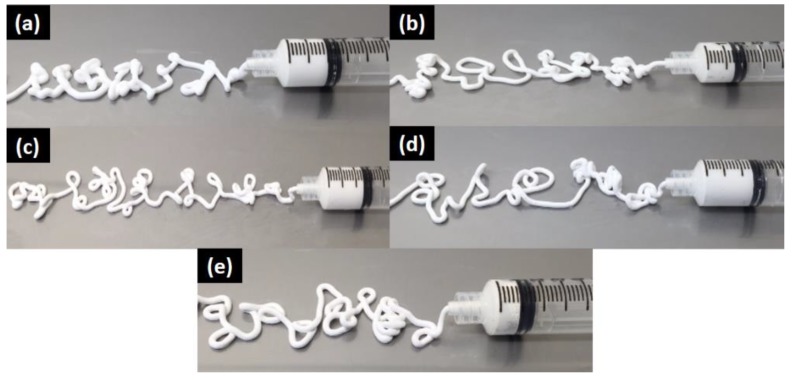
Injectability of smart CPC-x BT formulations where (**a**) x = 0 wt %, (**b**) x = 10 wt %, (**c**) x = 20 wt %, (**d**) x = 30 wt %, and (**e**) x = 40 wt %.

All CPC formulations with and without BT showed good injectability without any filter pressing. The injectability of smart CPC-x BT pastes are presented in Figure 6. All compositions retained their injectability up to 5–6 min after starting to mix the powders and colloidal silica.

### 3.6. Washout Resistance Test

**Figure 7:**
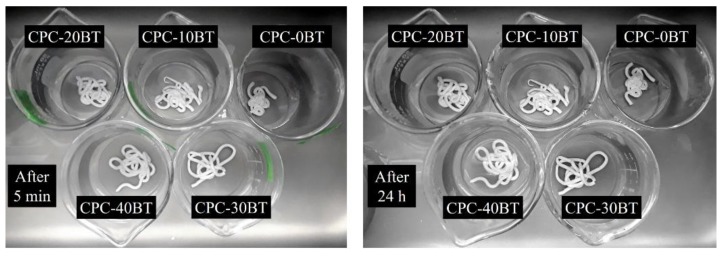
Washout resistance test of various CPC formulations in SLS.

All CPC formulations injected into SLS solution showed excellent resistance against deterioration in a harsh environment created by SLS. No signs of disintegration were observed after keeping those in SLS for 5 min. The images shown in Figure 7 show the absence of any degradation after 5 min signifying good washout resistance of the cement samples. The samples were held further in immersion for 24 h and after the specified time frame, no disintegration was observed. 

### 3.7. Biodegradation

**Figure 8:**
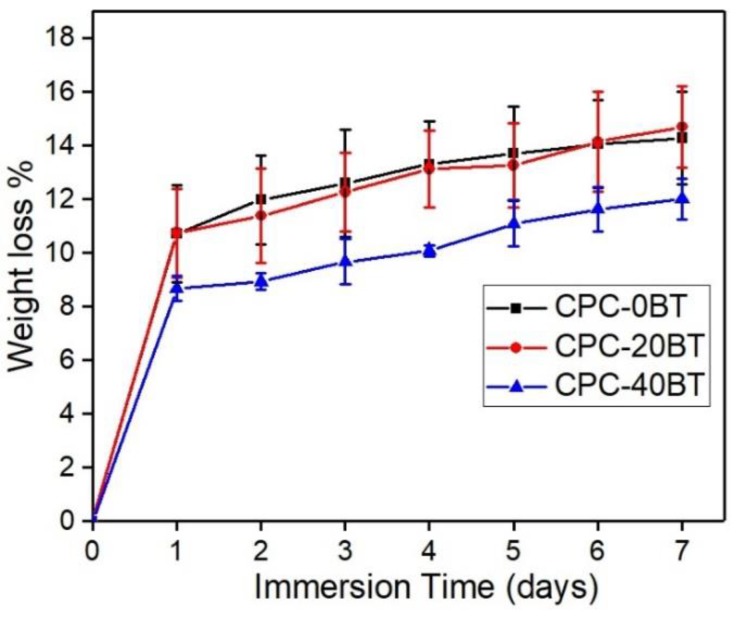
Weight loss% of CPC-x BT samples in 1.5 × t-SBF at different points of time.

Figure 8 shows the weight loss evaluation of various cement samples in vitro. The medium used for testing was 1.5 × t-SBF and the results are expressed in terms of weight loss %. For CPC samples with 0 and 20 wt % BT, weight loss rates are comparable with similar weight loss rate. Although no statistical difference was observed, 40 wt % samples showed comparatively less degradation rate as compared to 0 and 20 wt % samples.

### 3.8. Cytocompatibility

**Figure 9:**
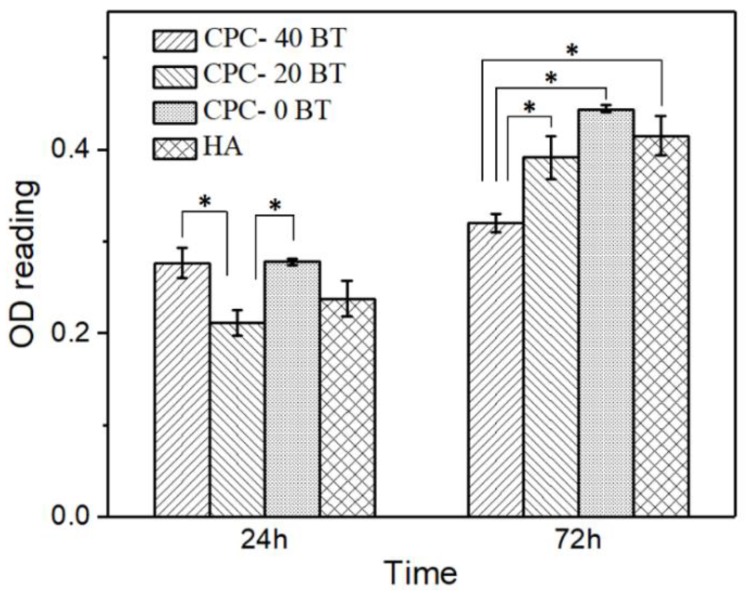
Optical density (OD) values of OB-6 pre-osteoblast cells seeded in extract of smart CPC-x BT formulations and HA for 24 h and 72 h, * refers to statistically different pair (*p* < 0.05).

The in vitro cell proliferation tests were carried out on 0, 20 and 40 wt % samples with pre-osteoblast OB-6 cells extracted from mice and the results are presented in Figure 9. Hydroxyapatite (HA) was used as a negative control for the cell studies. The number of cells is proportional to the optical density (OD) readings and thus the results are presented in terms of OD450 readings. After a 24 h period, the number of cells attached on CPC-20BT samples were observed to be lower (*p* < 0.05) as compared to CPC with 0 and 40 wt % samples. However, there were no significant difference (*p* > 0.05) in the number of cells attached when compared with the HA control. On the contrary, cell cultured with 40 wt % samples extracts showed significantly lower (*p* < 0.05) values than the rest of the CPC formulations and HA control extracts after a 72 h period. This implies that CPC-40 BT samples exhibited lower cytocompatibility with an increase of incubation time, as compared to CPC-20 BT, which helped in enhanced cell proliferation over 72 h.

**Figure 10:**
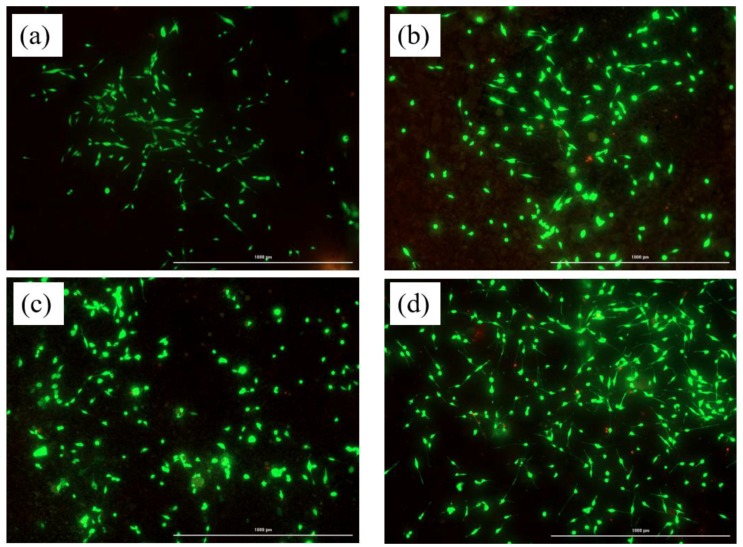
Live and dead cell assay for the cells attached and proliferating along the surface of (**a**) CPC-0 BT, (**b**) CPC-20 BT, (**c**) CPC-40 BT, and (**d**) HA surface at day 5. The viable cells are indicated by green fluorescence and dead cells by red fluorescence. Scale 1000 µm.

The results from live and dead assay are presented in Figure 10 where the viable and dead cells are represented by green and red fluorescence respectively. The CPC-0 BT showed a relatively lower number of cells attachment and proliferation than smart CPC formulations with BT. Moreover, CPC-20/40 BT and the HA control had a comparable number of live cells attached to their surfaces implying higher cell proliferation on BT incorporated samples. This section is divided into subheadings to provide a concise and precise description of the experimental results, their interpretation, as well as the experimental conclusions that can be drawn.

## 4. Discussion

Magnesium ion (Mg^2+^) is the second most abundant intracellular divalent cation and has been known for its involvement in diverse cellular functions. Since Mg^2+^ assists in bone mineral metabolism, formation and the crystallization process, in addition to the Ca^2+^ ion, premixed powder (PMP) has been integrated with magnesium hydroxide [Mg(OH)_2_] [43,44,45]. At the beginning of our study, setting time was measured by using PMP, MgO as powder phase and colloidal silica as a liquid phase. This composition completely set within 3 min and did not allow sufficient working time as the ideal handling requirements for an injectable CPC are: initial setting time 3–8 min, cohesion time ≥ 1 min, and final setting time ≤ 15 min [23,24]. The setting time can be modified by adjusting the cement composition and incorporating additives such as sodium orthophosphates, citric acid, gelatinized starch [24], borax or sodium borate decahydrate (Na_2_B_4_O_7_·10H_2_O) [46], p-chitosan [29], surface-modified multi-walled carbon nanotubes [42], and colloidal silica [38]. The present study uses borax as a setting time retardant. After numerous trials, the optimal concentration of borax was identified to be 4 wt % as this resulted in initial and final setting times of approximately 8 min and 15 min respectively (Table 4).

The setting mechanism of CPC is a continuous process and can be dissociated into two parts. First, the interaction between Ca^2+^ and Mg^2+^ with excess H_2_PO_4_^−^ and HPO_4_^2−^, forms a network to provide initial stability, and is responsible for initial cement setting [38]. The second part involves the hardening mechanism via entanglement of the precipitated crystals and thus results in the final setting [23,24]. The relevant chemical reactions involved during setting can be summarized as:

(2) $$\begin{array}{l} \mathrm{Ca}{\left(\mathrm{OH}\right)}_{2}+2H_{3}{\mathrm{PO}}_{4}+\mathrm{Mg}{\left(\mathrm{OH}\right)}_{2}+{\mathrm{Na}}^{+}\left(\mathrm{aq}\right)+{\mathrm{HCO}}_{3}{}^{-}\left(\mathrm{aq}\right)+\mathrm{Si}-\mathrm{OH}+\mathrm{Si}-\mathrm{OH}\\ \;\to {\mathrm{CaHPO}}_{4}\left(\mathrm{active}\right)+{\mathrm{MgHPO}}_{4}\cdot 3H_{2}O\left(\mathrm{active}\right)+2H_{2}O+{\mathrm{Na}}^{+}\left(\mathrm{aq}\right)\\ \;+{\mathrm{HCO}}_{3}{}^{-}\left(\mathrm{aq}\right)+\mathrm{Si}-O-\mathrm{Si} \end{array}$$

(3) $$\begin{array}{l} {\mathrm{CaHPO}}_{4}\left(\mathrm{active}\right)+{\mathrm{MgHPO}}_{4}\cdot 3H_{2}O\left(\mathrm{active}\right)+H_{2}O+\mathrm{Si}-\mathrm{OH}+\mathrm{Si}-\mathrm{OH}\\ \;\to {\mathrm{CaHPO}}_{4}\left(\mathrm{stabilized}\right)+{\mathrm{MgHPO}}_{4}\cdot 3H_{2}O\left(\mathrm{stabilized}\right)\\ \;+\mathrm{amorphous}\;\mathrm{phases}+2H_{2}O+\mathrm{Si}-O-\mathrm{Si} \end{array}$$

Generally, the infusion of BT within the cement samples lowers the overall concentration of active self-setting CaHPO_4_, accounting for reduced matrix entanglement which in turn increases the setting time. In addition, BT addition undermines the physical bond between plate-like monetite crystals resulting in reduced mechanical strength. Except 10 wt % BT samples, all the samples have trivial differences (*p* > 0.05) in the setting time values. Significant reduction of setting parameters for 10 wt % samples can be attributed to higher siloxane group formation (Figure 3) in these samples as indicated in our FTIR results. The self-hardening CPCs must exhibit a mechanical strength at least equivalent to trabecular bones i.e., 10 MPa [24]. All as-prepared CPCs displayed strength higher than 15 MPa proving their viability in orthopedic applications (Figure 1). Wolff et al. reported compressive strength of approximately 55 MPa for an injectable bone cement consisting of monocalcium phosphate monohydrate, tricalcium phosphate, and calcium carbonate mixed with sodium phosphate solution [47]. Whereas a comparative study carried out by Dadkhah et al. reported compressive strength of injectable calcium sulphate cement (CSC), Spine-Ghost cement and Cerament^®^ after 24 h to be 15.8 MPa, 14 MPa, and 8.2 MPa respectively. Comparing our data with Dadkhah et al., we can claim that our formulation is superior to CSS [48]. No detrimental effect on compressive strength occurs up to 40 wt % of BT addition. Up to 10.0 vol% (or 35 wt %) BT content filled within PMMA bone cements, R.G. Carrodeguas et al. [21] reported an insignificant effect on ultimate compressive strength of cement. The subunits of colloidal silica particles are usually un-joined Si(OH)_2_. Initially OH^−^ at the surface of colloidal silica particles assists in electrostatic dispersion of cement reactants among nanosilica particles [38,41]. The colloidal silica particles then interact together and polymerize into a 3D network of siloxane (Si–O–Si) groups forming a bond between as-produced DCPA, newberyite crystals, and incorporated BT simultaneously [41]. Therefore, colloidal silica condensation to siloxane via gelling mechanism [41] resisted the setting time increment and compressive strength decrement in our cement samples.

The prime constituent of PMP being Ca(OH)_2_ and H_3_PO_4_, the XRD analysis (Figure 2) shows major peaks for monetite. Also, due to the presence of Mg(OH)_2_ as a precursor, the newberyite peak was observed in the XRD results. Mg^2+^ and Ca^2+^ cations have similar chemical affinity; thus, formation of newberyite is anticipated. Newberyite is an important magnesium phosphate (MgP) compound which has shown promising properties in several orthopedic applications. Thus, its presence would not harm the bioceramic performance [49]. Besides, MgPs in general are slowly gaining attention in the literature as the CaO–P_2_O_5_ system [50]. Additional BT peaks in BT incorporated samples without any changes in the phases and crystal structure implies there is no undergoing chemical reaction involving BT in the samples. Our reaction system has a molar ratio of (Ca + 0.5Na)/P less than 1 (i.e., excess phosphoric acid), and consist of Ca^2+^, Mg^2+,^ and Na^+^ cations. Yet, XRD results did not show peaks of other phases such as Ca(H_2_PO_4_)_2_, Mg(H_2_PO_4_)_2_, NaH_2_PO_4_. Since microwave synthesis favors amorphous phases, other above mentioned phases might have formed amorphous compounds by partial transformation of H_2_PO_4_^−^ and HPO_4_^2−^ ions with the available cations [51,52]. The SEM images (Figure 4) revealed the presence of large plate-like monetite crystals. The distribution of plate like crystals can be seen throughout the surface of 10 and 20 wt % BT samples. The plate-like monetite crystals have a capability of forming a network easily by interacting with each other. This network accelerates the initial stabilization of the cement and reduces the solidification time. Thus, although insignificant the 10–20 wt % of BT addition led towards a lower initial setting time. Furthermore, the surface morphology at higher magnification (Figure 4f) confirmed the infusion of BT within the CPC structure. 

Incorporation of ferroelectric BT contributes towards the enhancement of the electrical properties of formulation simulating the actual bone scenario. Under stress conditions, BT generates electrical charges on the sample surfaces. Negative charges on the sample surface adsorbs Ca^2+^ cations which act as nuclei for the formation of the Ca–P layer [53]. Ca^2+^ cations also facilitate protein adhesion such as intergrins, fibronection, and osteonectin. On the other hand, limited apatite formation is expected from positive charges on the surface as they attract antiadhesive anionic groups such as HPO_4_^2−^ and HCO_3_^2−^ [54]. There are instances in the literature where apatite formation was accelerated by using a higher concentration of SBF [55]. The colloidal silica is also responsible for apatite nucleation and growth [56]. Even though the increment of apatite on SBF immersed BT incorporated samples was insignificant, these samples under in vivo stress conditions are expected to increase apatite formation as compared to CPC-0 BT. 

As we are dealing with injectable cement, the more realistic evaluation of washout resistance is by injecting cement paste into SLS straight after loading into the syringe and therefore the tests were carried out following this approach. Magnesia, MgO, enhances the anti-washout property of calcium phosphate cements. Moreover, the binding property of silica with CPCs and BT is also expected to improve the washout resistance. The washout resistances of monetite cement with and without chitosan in SLS as reported by Touny et al. [40] were 1 h and immediate dissociation into powder respectively. Our cement formulations have better washout resistance as all formulation maintained their shape even after 24 h (Figure 7). 

Bone graft cements demand a combination of good injectability and proper hardness to minimize invasiveness of surgery [24]. It is noteworthy that the as-prepared CPCs formulations maintained proper injectability up to 5–6 min along with high workability and cohesion time. When compared to the commercially available Norian SRS^®^ cements, present CPC formulation displayed superiority in terms of injectability. Norian SRS^®^ cements exhibit filter pressing with 1.5 mL uninjectable cement out of 4.5 mL [24] whereas with 5 mL of our formulation paste inside the syringe, each of our compositions avoided this phenomenon. More often addition of fillers in CPCs reduces porosity causing slower resorption, slow bone substitution, poor injectability and other poor rheological properties [24]. On the contrary, in our work, the addition of BT as filler is promoting the bone substitution rate, while the injectability and degradation rate remained unaffected. However, the present CPC formulations have some porosity. The presence of pores in the formulations is mainly because of trapped air. It should also be noted that porosity, pore size, and interconnectivity of pores are important for cell penetration and bone ingrowth. R.G. Carrodeguas et al. [22] reported acceptable setting parameters, compressive strength, radiopacity, and injectability for surgical procedures with 20–50 wt % of untreated or silanated BaTiO_3_ or SrTiO_3_ in acrylic bone cements. The addition of 20–40 wt % BT into our CaP structure is expected to provide sufficient radiopacity to the cement under fluoroscopy. While considering the biodegradability aspect, BT ≤ 20 wt % in cement samples is more favorable. 

In vitro cytocompatibility tests are crucial preliminary tests for biomaterials before moving forward to more complex and expensive in vivo experiments. Incorporating the right amount of dopant leads to multi-functionality in various orthopedic materials. Recently our group doped Ag into CaPs and MgPs to make antibacterial coatings using microwave irradiation [57,58]. The WST-1 assay OD reading after 24 h revealed lowered number of cell attachment on 20 wt % samples than for 0 and 40 wt % samples, however, they were comparable to the HA control. The biocompatibility behavior of particulate filled composite can be different from its identical bulk composite [19]. This can be the plausible reason behind the low OD reading of 20 wt % samples as compared with 0 wt % samples. As a surprise, the initially inhibiting 20 wt % samples showed OD reading comparable (*p* > 0.05) to 0 wt % samples and HA control after 72 h period. Fine (<2 µm) wear particles can translocate to the different organs via systemic circulation and lead to cell toxicity [19]. BT used in this study had a particles size of less than 2 µm. Therefore, the reduced OD values for 40 wt % samples after 72 h period can be due to the presence of excessive fine BT particles. This clearly indicates CPC-20 BT to be the optimum composition for biocompatible ferroelectric CPC cement. All the samples showed higher OD readings compared to the 24 h period implying cell proliferation on CPC-x BT samples. Furthermore, the live and dead cell assay after 5 days incubation clarified the non-toxic nature of cement samples incorporated with BT. Higher cell proliferation on CPC-20/40 BT sample as compared to CPC-0 BT thus implies the enhancement of earlier stage >osteogenesis.

## 5. Conclusions

Smart CaP cements incorporated with ferroelectric BT were synthesized using microwave energy and colloidal silica. The as-prepared CPCs were bioactive, biocompatible, and most importantly bio-degradable. All cement formulation behaved in a similar fashion regarding setting time, mechanical strength, bioactivity, injectability, and washout resistance. Moreover, based on the cytocompatibility and biodegradation rate, we conclude the most favorable CPC composition for better cell viability and biodegradation to be 20 wt % of BT. These promising physical, mechanical and biological characteristics of novel injectable piezoelectric CPCs makes them possible candidates for a new generation smart biodegradable CaP bone cement.

## Figures and Tables

**Table 1:** Information on incorporated colloidal silica.

Product	Concentration	Surface Area	pH	Density
LUDOX^®^ HS-40 colloidal silica	40 wt % suspension in H_2_O	~220 m^2^/g	9.8	3 gm/mL at 25 °C

**Table 2:** Different smart CaP cement compositions.

Cement	PMP (g)	Borax (g)	MgO (g)	BaTiO_3_ (g)	Colloidal Silica (mL)
CPC-0 BT	5	0.20	0.25	-	1.910
CPC-10 BT	5	0.20	0.25	0.545	2.100
CPC-20 BT	5	0.20	0.25	1.090	2.290
CPC-30 BT	5	0.20	0.25	1.635	2.480
CPC-40 BT	5	0.20	0.25	2.180	2.670

**Table 3:** Chemical composition of 1.5 × t-SBF.

Order	Reagent	Amount (Weight g per L or Volume mL)
1	NaCl	9.8184
2	NaHCO_3_	3.4023
3	KCl	0.5591
4	Na_2_HPO_4_	0.2129
5	MgCl_2_·6H_2_O	0.4574
6	1M HCl	15 mL
7	CaCl_2_·2H_2_O	0.5513
8	Na_2_SO_4_	0.1065
9	TRIS	9.0855
10	1M HCl	50 mL

**Table 4:** Setting time for smart CPCs with various wt % barium titanate (BT).

Cement	Initial Setting Time (min)	Final Setting Time (min)
CPC-0 BT	8.83 ± 0.72	15.33 ± 0.24
CPC-10 BT	7.58 ± 0.12	13.67 ± 0.62 *
CPC-20 BT	7.92 ± 0.42	15.17 ± 0.51
CPC-30 BT	8.08 ± 0.72	16.33 ± 0.24
CPC-40 BT	8.50 ± 0.35	15.92 ± 0.42

* represents significantly different from rest of the composition.
